# A Tool for Segmentation of Secondary Structures in 3D Cryo-EM Density Map Components Using Deep Convolutional Neural Networks

**DOI:** 10.3389/fbinf.2021.710119

**Published:** 2021-11-03

**Authors:** Yongcheng Mu, Salim Sazzed, Maytha Alshammari, Jiangwen Sun, Jing He

**Affiliations:** Department of Computer Science, Old Dominion University, Norfolk, VA, United States

**Keywords:** 3-dimensional (3D), image, neural networks, convolutional, protein, secondary structure, cryo-electron microscopy, deep learning

## Abstract

Although cryo-electron microscopy (cryo-EM) has been successfully used to derive atomic structures for many proteins, it is still challenging to derive atomic structures when the resolution of cryo-EM density maps is in the medium resolution range, such as 5–10 Å. Detection of protein secondary structures, such as helices and β-sheets, from cryo-EM density maps provides constraints for deriving atomic structures from such maps. As more deep learning methodologies are being developed for solving various molecular problems, effective tools are needed for users to access them. We have developed an effective software bundle, DeepSSETracer, for the detection of protein secondary structure from cryo-EM component maps in medium resolution. The bundle contains the network architecture and a U-Net model trained with a curriculum and gradient of episodic memory (GEM). The bundle integrates the deep neural network with the visualization capacity provided in ChimeraX. Using a Linux server that is remotely accessed by Windows users, it takes about 6 s on one CPU and one GPU for the trained deep neural network to detect secondary structures in a cryo-EM component map containing 446 amino acids. A test using 28 chain components of cryo-EM maps shows overall residue-level F1 scores of 0.72 and 0.65 to detect helices and β-sheets, respectively. Although deep learning applications are built on software frameworks, such as PyTorch and Tensorflow, our pioneer work here shows that integration of deep learning applications with ChimeraX is a promising and effective approach. Our experiments show that the F1 score measured at the residue level is an effective evaluation of secondary structure detection for individual classes. The test using 28 cryo-EM component maps shows that DeepSSETracer detects β-sheets more accurately than Emap2sec+, with a weighted average residue-level F1 score of 0.65 and 0.42, respectively. It also shows that Emap2sec+ detects helices more accurately than DeepSSETracer with a weighted average residue-level F1 score of 0.77 and 0.72 respectively.

## Introduction

Although many atomic structures have been resolved from cryo-EM density maps with a resolution of 4 Å or higher, deriving atomic structures from cryo-electron microcopy (cryo-EM) with medium resolution (5–10 Å) is challenging due to quality of density maps in this resolution range. With the rapid improvement of resolution in cryo-electron tomography maps and in subtomogram averaging, more density maps are expected to reach the medium resolution for interpretation. In some cases, certain components of an entire density map may show lower resolution than most other components, due to potential experimental and computational artifacts and flexibility of molecules in certain regions of a molecular complex. For cryo-EM density maps with the medium resolution, density features of a protein backbone are often not distinguishable. It is generally hard to distinguish the location of α-carbons of a protein backbone from such images. Our understanding about medium-resolution density maps is currently limited by the availability of atomic structures. Almost all atomic structures derived from medium-resolution cryo-EM maps are based on fitting template atomic structures of the Protein Data Bank (PDB). Flexible fitting (Chapman, 1995; Wriggers et al., 2000; Chen et al., 2001; Wriggers and Birmanns, 2001; Trabuco et al., 2009) and rigid-body fitting (Cowtan, 2010) are two types of modeling methods to derive atomic structure from medium-resolution maps. When no suitable templates are available, various attempts have been made to utilize protein secondary structure information. To establish an initial trace of a backbone, a critical step is to map secondary structures of a protein sequence to their locations in the cryo-EM density map; this is a step also referred to as finding the topology of secondary structures (Abeysinghe et al., 2008; Al Nasr et al., 2014; Biswas et al., 2016). Many methods, such as JPred and SSpro, are available to predict sequence segments of protein secondary structures (Cole et al., 2008; Magnan and Baldi, 2014). Since secondary structures, such as α-helices and β-sheets, have density characteristics, they are distinguishable in density maps at the medium resolution. Location of α-helices and β-sheets in a density map provides constraints about the atomic structure of the protein.

There are three broad categories of methods for detection of secondary structures, and we use the detection of helices from medium-resolution maps to discuss the trend of development. The first generation of methods use image processing techniques to detect the cylindrical character of helix density (Jiang et al., 2001; Dal Palù et al., 2006; Baker et al., 2007; Rusu and Wriggers, 2012; Si and He, 2013). The second generation of methods use machine learning ideas to measure multiple features of helices (Ma et al., 2011; Si et al., 2012). The third generation of methods utilize deep learning and availability of large set of density maps in Electron Microscopy Data Bank (EMDB) (Li et al., 2016; Maddhuri Venkata Subramaniya et al., 2019; Wang et al., 2021). However, precise detection of secondary structures from medium-resolution density maps is still challenging. Short helices are, in general, harder to be detected than longer helices. Detection of β-sheets is generally more challenging than detection of α-helices. In addition to challenges in detection of subtle differences in various shapes, the variety of data quality in deposited density maps presents a major challenge for accurate detection of secondary structures (Wriggers and He, 2015; Sazzed et al., 2020).

Although various methodologies have been proposed, there are limited tools for detection of secondary structures from cryo-EM density maps with medium resolution. Existing tools for detection of helices and β-sheets, such as SSEhunter (Baker et al., 2007), SSETracer (Si and He, 2013), are image-processing based methods that are often dependent on user-selected parameters. Emap2sec+ (Wang et al., 2021) is a deep learning method, but users are required to go through multiple steps to install software libraries with certain dependencies. Although recent deep-learning approaches show improved detection of secondary structures from medium-resolution maps, there has not been a tool available for users.

Chimera and ChimeraX are popular visualization platforms for molecular images and structures (Pettersen et al., 2004; Pettersen et al., 2021). Various software plugins have been developed for Chimera and ChimeraX. ChimeraX uses a Toolshed mechanism for user-developed applications to be integrated. However, most such applications are less dependent on other large software platforms. None of them involves a deep-learning framework. Deep-learning-based programs require frameworks such as Tensorflow (Abadi et al., 2016) and PyTorch (Paszke et al., 2019), which support the use of GPUs. It is not clear how effective they can be integrated with molecular visualization platforms, such as ChimeraX. In this paper, we propose a tool, DeepSSETracer, for secondary structure detection from cryo-EM density component maps using a convolutional neural network. The design of DeepSSETracer software bundle applies to components of a cryo-EM density map with the maximum size of 100 voxels in any of the three dimensions. Using a data set containing the density maps of 28 protein chains, DeepSSETracer bundle shows an overall residue-level F1-scores of 0.72 and 0.65 for detection of helices and β-sheets, respectively. Our experiments show that it takes about 6 s to load the pre-trained model and to complete the predicted labels of secondary structures on one CPU and one GPU of a Linux server. However, initialization of image data into the convolutional neural network takes about 26 s, for which optimization is needed in future.

## Results and Discussion

### DeepSSETracer Bundle for ChimeraX

**FIGURE 1:**
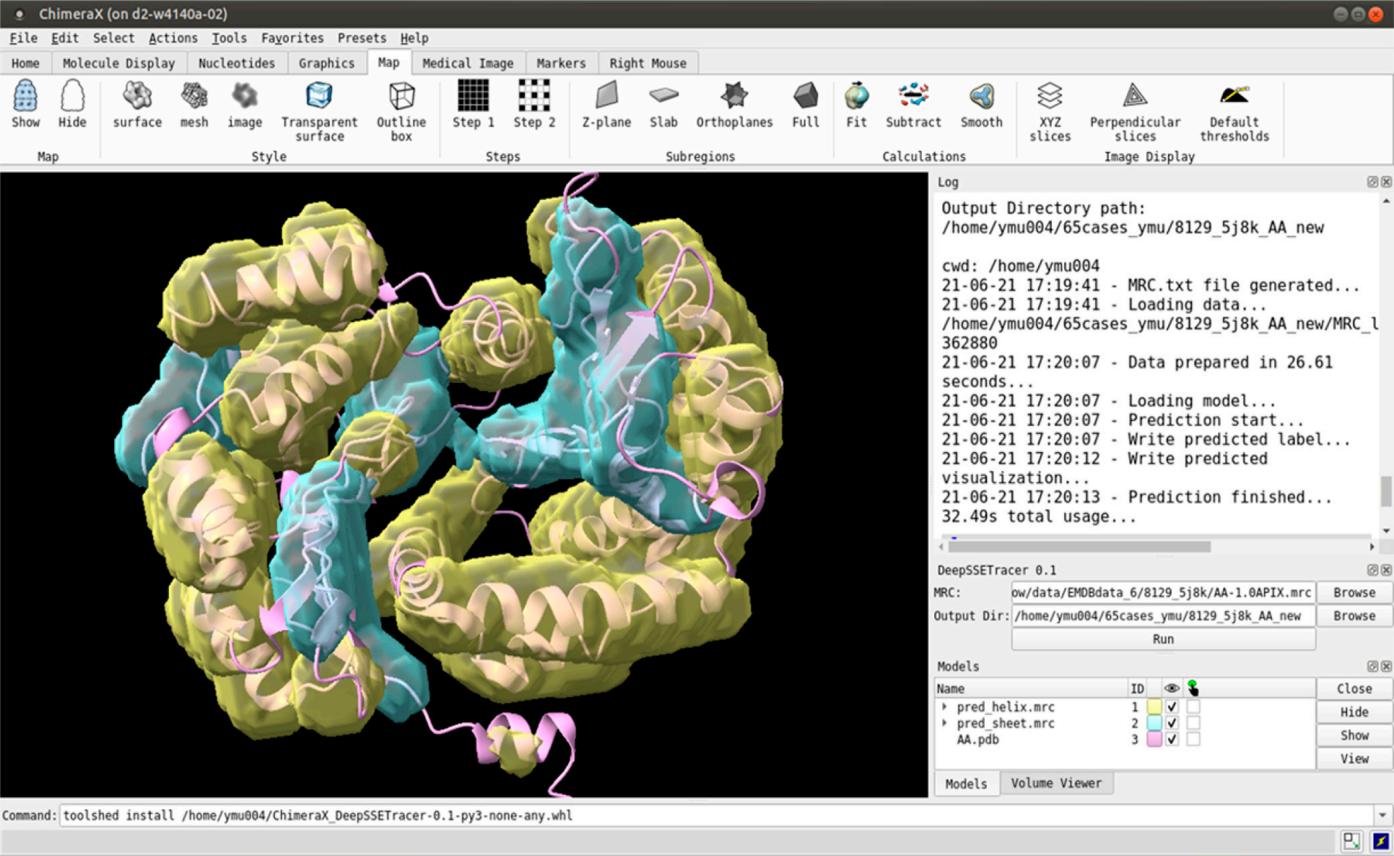
A snapshot of the bundle plugin DeepSSETracer 0.1 installed on ChimeraX. The helix voxels (yellow) and β-sheet voxels (cyan) were detected using DeepSSETracer bundle in ChimeraX.

The DeepSSETracer method was packaged into a bundle that can be installed in ChimeraX as a *Python* wheel (Pettersen et al., 2021). The deep neural network and a pre-trained model were packaged in a wheel file. Once a wheel file is created, a user can install it using ChimeraX command “toolshed install” to add it under Tools (Figure 1 bottom). The DeepSSETracer GUI accepts as input a density map in MRC format with a 1 Å per voxel sampling (in the right panel of Figure 1). The output contains two image files in MRC format, one for detected helix and one for β-sheets. The current design is for a component of a density map with a maximum size of 100 voxels in any of the three dimensions. Figure 1 provides an example of detected secondary structure regions shown in ChimeraX. The input cryo-EM component map corresponds to the chain AA of 5j8k (PDB ID), with 446 amino acids in length. DeepSSETracer was trained using the GEM-Unet method (details in Materials and Methods) (Deng et al., 2020). No user-given parameters are needed.

In order to evaluate the effectiveness of DeepSSETracer as a tool that can be completed in reasonable time for a user, run-time performance was recorded for five cases with different sizes of maps (Table 1 column 3 and 4). Time was measured from a Linux system with ChimeraX installed. The Linux system has a typical setup of X11 forwarding for graphics display. It contains an X11 client where ChimeraX is installed and an X11 server as a login node. The server/login node runs a modified X11 server to communicate between the server and the client. The server accepts requests from a user desktop or laptop that runs a Windows Remote Desktop to connect to the server. The server/login node passes graphics display from the X11 client to the user through Windows Remote Desktop protocol. The Linux system contains Intel Xeon(R) Gold 6,130 @ 2.1 GHz (32 slots) CPU and AVX512, four x Nvidia Tesla V100 GPU. The time measurement in Table 2 corresponds to the use of one CPU and one GPU in each case. A small test set of five cases was used to monitor the time used in several steps of the performance. The five cases contain protein chains with 52 amino acids to 446 amino acids, ranging from sizes of 57 × 47 × 42 = 112,518 voxels to 93 × 62 × 66 = 380,556 voxels in the input component maps (Table 1). The major time consumed is at the initialization of the input 3D image. As an example, it took 26.10 s for the component image of 3850 (EMDB ID) that corresponds to Chain four of 5oqm (PDB ID) in the step of data initialization that includes loading the 3D image of MRC format, density normalization, and padding the image. Once the initialization is done, the 3D image is passed to the CNN network for prediction of secondary structures. The step for the CNN architecture to perform prediction and output results only takes 6.12 s for the largest of the five cases (Table 1). Our experiments show that our five-layer U-Net only took about 6 s to perform the computation once data were initialized. The current design of data initialization took about four times longer than the actual computation in the network. Our current design uses standard data input mechanisms provided by PyTorch. It is possible to speed up the initialization step with custom-built code to handle the need of specific types of 3D images used in the cryo-EM community. Although the training of DeepSSETracer took many hours, using the trained model for prediction completed in reasonable amount of time. The tool is potentially applicable for typical desktops and laptops, particularly those with GPU support.

**TABLE 1:** Run time of DeepSSETracer 0.1 on a Linux System. From left to right: EMDB ID, PDB ID, chain ID, the number of Cα atoms in the chain for H: helix, S: β-sheet, T: entire chain, the number of voxels in each of the X, Y, Z dimensions, the time to load and initialize data, and the time for CNN network to produce the results using one CPU and one GPU on a Linux server.

EMDB_PDB_Chain	Cα (H/S/T)	Size (X, Y, Z)	Data initialization (second)	CNN (second)
3850_5oqm_4	128/49/297	93, 62, 66	26.10	6.12
8129_5j8k_AA	187/60/446	74, 59, 83	27.08	5.81
8129_5j8k_D	170/41/384	74, 78, 64	26.24	5.84
1657_4v5h_AE	42/38/150	52, 77, 57	16.60	3.64
5943_3j6y_80	12/8/52	57, 47, 42	9.00	1.92

**TABLE 2:** Evaluation of detected helices and β-sheets from DeepSSETracer 0.1 and Emap2sec+ using 28 cryo-EM component maps. From the left to right: the EMDB ID, PDB ID, chain ID with the resolution of the cryo-EM map in parentheses, the number of Cα atoms in the STRIDE-annotated PDB file for H: helix, S: β-sheet, T: total of the chain, voxel-level of F1-scores for helix detection (H-V) and β-sheet detection (S-V), residue-level F1-scores for helix detection (H-R) and β-sheet detection (S-R) for DeepSSETracer 0.1 and Emap2sec+ respectively. NA: zero number of residues of the corresponding class in the atomic structure and undefined precision or recall.

EMDB_PDB_Chain (resolution Å)	Cα(H/S/T)	DeepSSETracer 0.1				Emap2sec+	
		H-V	S-V	H-R	S-R	H-R	S-R
1798_4v5m_AE (7.80)	42/58/150	0.64	0.54	0.85	0.66	0.86	0.59
2994_5a21_G (7.20)	37/21/133	0.38	0.37	0.37	0.41	0.43	0.31
3206_5fl2_K (6.20)	12/47/106	0.62	0.62	0.8	0.78	0.25	0
3491_5mdx_H (5.30)	33/0/42	0.69	NA	0.84	NA	0.79	NA
3850_5oqm_4 (5.80)	128/49/297	0.58	0.59	0.71	0.73	0.73	0.67
3850_5oqm_g (5.80)	81/0/85	0.74	NA	0.98	NA	0.98	NA
3948_6esg_B (5.40)	51/0/78	0.70	NA	0.81	NA	0.97	NA
4041_5ldx_I (5.60)	48/19/176	0.51	0.39	0.69	0.56	0.61	0.43
4078_5lms_D (5.10)	86/18/208	0.52	0.32	0.63	0.38	0.65	0.53
4141_5m1s_B (6.70)	81/158/366	0.56	0.55	0.72	0.73	0.68	0.69
4182_6f42_G (5.50)	16/66/180	0.55	0.46	0.70	0.71	0.75	0.57
5942_3j6x_25 (6.10)	25/5/70	0.18	0.15	0.14	0.27	0.71	0
5943_3j6y_80 (6.10)	12/8/52	0.44	0	0.61	0	0.51	0
6149_3j8g_W (5.00)	19/48/94	0	0.59	0	0.77	0.59	0
6446_3jbi_V (8.50)	116/0/131	0.72	NA	0.87	NA	0.92	NA
6456_3jbn_AL (6.70)	89/8/211	0.57	0.18	0.75	0.29	0.83	0
6810_5y5x_H (5.00)	38/10/100	0.11	0.47	0.14	0.60	0.58	0
7454_6d84_S (6.72)	65/34/142	0.49	0.51	0.55	0.64	0.66	0.27
8016_5gar_O (6.40)	65/0/80	0.26	NA	0.24	NA	0.7	NA
8128_5j7y_K (6.70)	71/0/93	0.72	NA	0.89	NA	0.92	NA
8129_5j8k_AA (7.80)	187/60/446	0.62	0.48	0.75	0.63	0.73	0.23
8129_5j8k_D (7.80)	170/41/384	0.58	0.29	0.69	0.38	0.73	0
8130_5j4z_B (5.80)	63/9/154	0.73	0.28	0.88	0.33	0.79	0.44
8135_5iya_E (5.40)	88/44/210	0.64	0.52	0.79	0.67	0.79	0.68
8357_5t4o_L (6.90)	106/0/160	0.67	NA	0.82	NA	0.82	NA
8518_5u8s_A (6.10)	114/13/208	0.69	0.54	0.86	0.68	0.88	0.33
8693_5viy_A (6.20)	68/0/133	0.66	NA	0.83	NA	0.75	NA
9534_5gpn_Ae (5.40)	59/0/88	0.60	NA	0.75	NA	0.83	NA
**Weighted Average**	**1970/716/4,577**	**0.59**	**0.49**	**0.72**	**0.65**	**0.77**	**0.42**

Weighted average: averaged F1 scores of all 28 test cases, weighted by the ratio of the number of residues of the class in each case and the total number of residues in the test set.

### Evaluation of Secondary Structure Detection Using F1-Scores

The performance of DeepSSETracer was evaluated by computing the F1-scores. The F1-score is known as a metric providing less biased measurement comparing to accuracy when there is considerable imbalance in the class distribution of the data. Two levels of F1-scores were calculated respectively for the prediction of individual voxel labels and of residual labels in the protein chains. The prediction for each residue was made by majority voting among the predicted labels of voxels within 3 Å radius of the Cα atom of the residue. In case of ties in voting, the priority is given to helix followed by sheet and then other. The true label of residues was determined by the secondary structure annotated using STRIDE (Frishman and Argos, 1995). For detailed evaluation, the F1-scores associated with predicting helix and β-sheet were computed separately.

**FIGURE 2:**
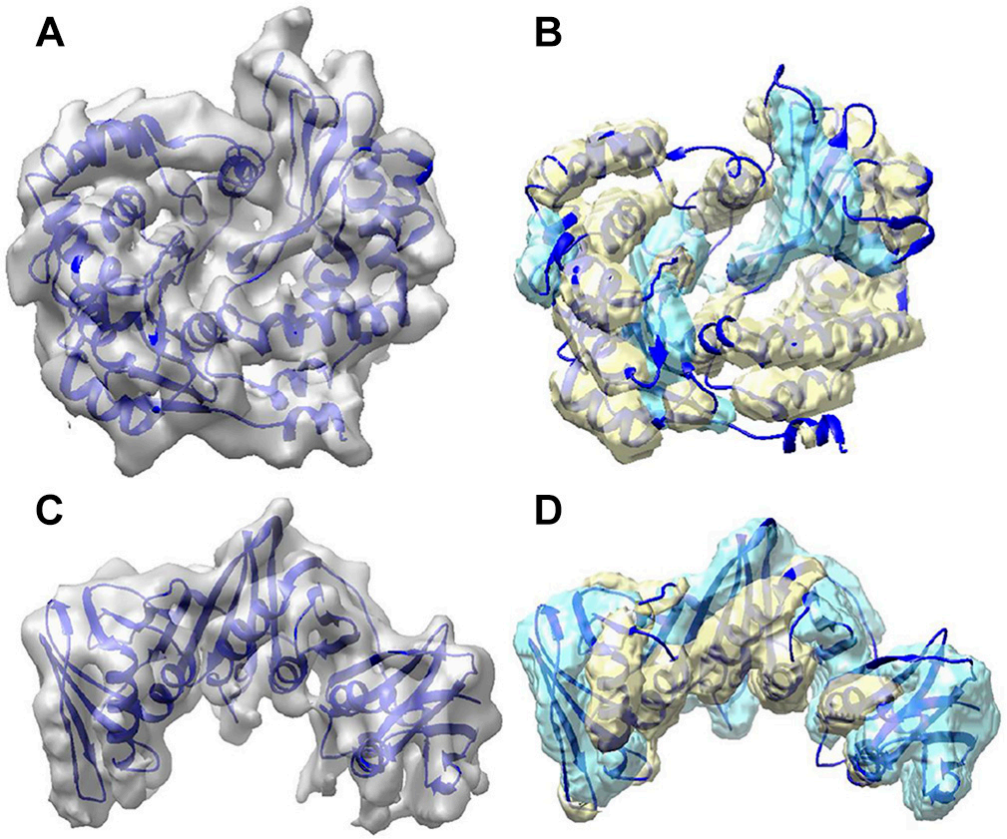
Segmentation of secondary structures using DeepSSETracer 0.1 for two chains. **(A)** The cryo-EM density map (EMD-8129, grey) component corresponding to the atomic structure of chain AA of 5j8k (PDB ID, blue ribbon). **(B)** Helix regions (yellow) and β-sheet regions (cyan) that were detected from the component map in (A) are overlayed with the atomic structure. **(C)** The cryo-EM density map (EMD-4141, grey) component corresponding to the atomic structure of chain B of 5m1s (PDB ID, blue ribbon). **(D)** Helix regions (yellow) and β-sheet regions (cyan) that were detected from the component map in (C) using DeepSSETracer 0.1. The display was created using Chimera (Pettersen et al., 2004).

Figure 2 shows the secondary structure detected using DeepSSETracer for two examples of component density maps with distinct distribution of helix and β-sheet voxels. In the first example (top row), the detection was made for the component of the cryo-EM density map (EMD-8129) that contains chain AA of 5j8k (PDB ID). The entire cryo-EM density map has 7.8 Å resolution, and a measure of cylindrical fit for helices classifies the component map in Bin 2, the second quality bin of the training data (Sazzed et al., 2020). This chain consists of 187 helix and 60 β-sheet Cα atoms (i.e., more helix voxels than β-sheet) that was annotated using STRIDE method (Frishman and Argos, 1995). The voxel-level F1-scores in this case are 0.62 for helix and 0.48 for β-sheet; while the residue based F1-scores are much higher for both, 0.75 and 0.63, respectively. The second example (bottom row) is the component of EMD-4141 corresponding to the chain B of PDB-5m1s that contains 81 helix and 158 β-sheet Cα atoms (i.e., more β-sheet voxels than helix). The voxel level F1-score for helix is 0.56, which is lower comparing to that in the first example, for β-sheet is 0.55 that is higher than that in the first example. The residue-level F1-score presents the similarly pattern, 0.72 for helix (reduced) and 0.73 for β-sheet (improved), with the reduction for helix prediction relatively small and much more significant improvement for β-sheet. In both cases, most portion of the helices and β-sheets in the chain was detected using DeepSSETracer (Figure 2). Results also suggest that it is, in general, easier to detect larger β-sheets and longer helices than those small secondary structures.

A set of 28 cases for testing was selected to avoid significant sequence similarity or structural similarity with any of the training data (see Materials and Methods). The resolution of the cryo-EM density maps ranges from 5 Å to 7.8 Å, and the length of the chains of the component maps is between 42 and 466 amino acids. Since different test cases contain different number of α-helices and β-sheets, a weighted average of the 28 cases was calculated, in which each case is weighted proportional to the number of helix/β-sheet residues of each case. For example, the result of a case contributes less if it contains only a small helix compared to a case with 10 helices when the weighted average for helix detection is calculated. The weighted average F1-score for helix detection is 0.59 at the voxel-level and 0.72 at the residue level. Residue-level F1-score is higher than voxel-level for both helix and β-sheet predictions on all the testing cases. Since a residue label is determined from majority voting of voxels in the neighborhood of the residue, it is intuitively less affected by mistakes from the minority of voxels. Therefore, F1-scores at the residue-level provides a more stable evaluation of the detection. Note that the F1-score characterizes different aspects of the results than accuracy. Unlike the accuracy, the F1-socre is more directly affected by false positives and false negatives. The residue-level F1-score may be used as a convenient evaluation for the detection of an individual class, such as helix or β-sheet. The DeepSSETracer predicts helices better than β-sheets, for which the weighted average of voxel and residue based F1-scores are 0.49 and 0.65, respectively.

### Analysis of Detection Results From DeepSSETracer and Emap2sec+

**FIGURE 3:**
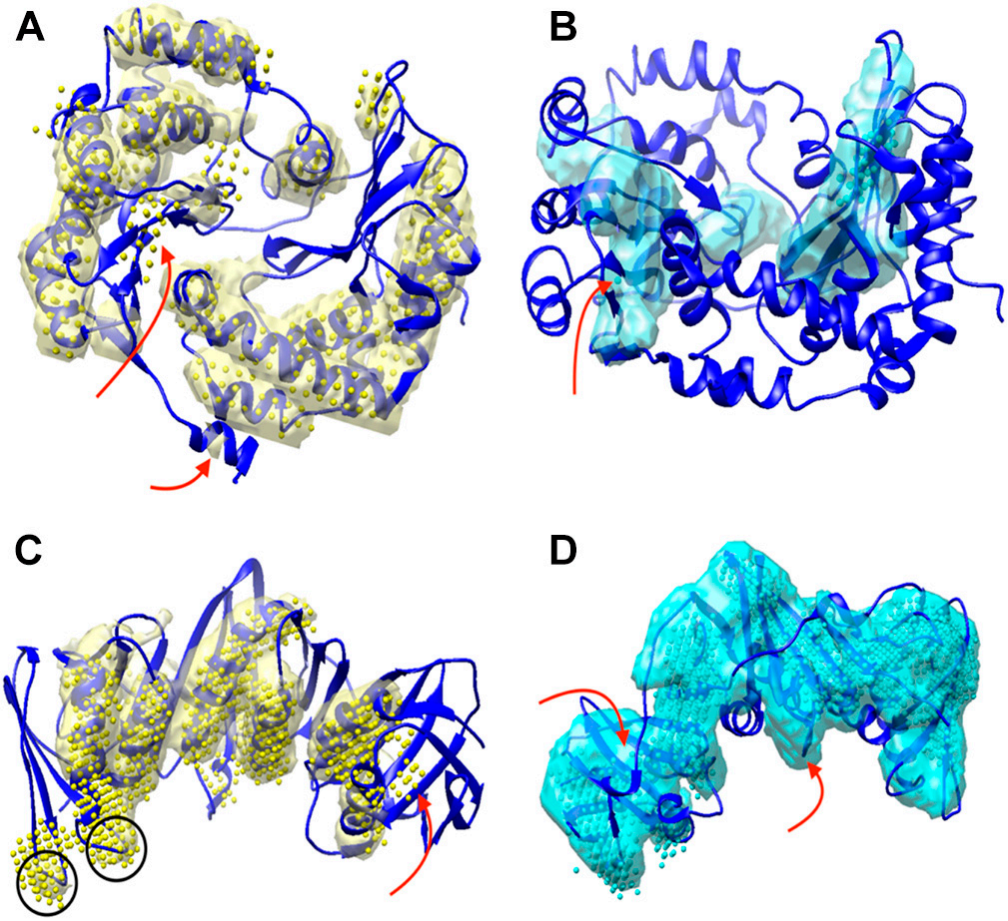
Secondary structures detected using DeepSSETracer 0.1 and Emap2sec+ for two chains. Two views of detected secondary structures in cryo-EM component map (EMD-8129, chain AA of PDB-5j8k (blue ribbon)) are shown for detected helix regions using DeepSSETracer 0.1 (yellow transparent volume) and Emap2sec+ (yellow dots) in **(A)** and β-sheet regions using DeepSSETracer (cyan transparent volume) and Emaps2sec+ (cyan dots) in **(B)**. The front view and the back view of the detected secondary structures from cryo-EM component map (EMD-4141, chain B of PDB-5m1s (blue ribbon)) using DeepSSETracer 0.1 (transparent volume) and Emap2sec+ (dots). Locations of some major differences in the detection are indicated with red arrows. Examples of false positive regions shared by both methods are indicated with black circles.

The detection of helices and β-sheets was compared between DeepSSETracer 0.1 and Emap2sec+ using the test set of 28 cryo-EM chain components. The Emap2sec+(Wang et al., 2021) program and instruction were downloaded from GitHub (https://github.com/kiharalab/Emap2secPlus), and the pre-trained models were downloaded from https://kiharalab.org/emsuites/emap2secplus_model/ in August 2021. The model used in the test is the 4-fold model that was an aggregated model, in which prediction was done by majority voting of predicted probabilities from the four networks. The density contours were obtained in EMDB and were used as suggested in the instruction of Emap2sec+. Specific parameters for running Emap2sec+ are as following: mode = 2 (a voted results from the four networks), type = 3 (experimental map), class = 3, contour = [EMDB contour]. Since all cryo-EM chain components are resized to 1 Å per voxel, there is no resizing needed when running Emap2sec+. The detected secondary structures using DeepSSETracer and Emap2sec+ were shown for two examples (Figure 3). Chain AA (author annotated ID) of PDB-5j8k, also annotated as chain CB on PDB website, has 446 amino acids. The detected helices from the two methods (yellow volume and yellow dots) are mostly co-located, with differences at some regions noted using red arrows (Figure 3A). The visually similar level of detection is shown from the residue-level helix F1-score of 0.75 and 0.73 for DeepSSETracer and Emap2sec+ respectively in this case (Table 2). The slightly lower F1-score of Emap2sec+ might reflect the main difference in its false positive detection of helix at a region of a β-sheet (the upper red arrow of Figure 3A). The main difference in this case is about the detection of β-sheets. Emap2sec+ detected only one dot (red arrow in Figure 3B) for the β-sheet on the left. This region contains eight segments of β-strands, four of which are segments 15–18, 34–36, 100–102, and 196–201 of the amino acid sequence. Although some segments are connected by loops that roughly parallel to neighboring strands, the loop segments can be indistinguishable from β-strand segments at the medium resolution. DeepSSETracer detected most portions of this β-sheet (cyan transparent), although some over-detection was noticed at the top the β-sheet seen in Figure 3B. The F1-score for β-sheet detection is 0.63 and 0.23 respectively for DeepSSETracer and Emap2sec+ (Table 2). The main contributor for the lower β-sheet F1-score for Emap2sec+ is likely to be the false negatives mostly in the β-sheet on the left but also some on the right β-sheet (Figure 3B). We observed that both methods made mistakes in similar regions, such as for the case of chain B of PDB-5m1s (black circles in Figure 3C), where turns were wrongly detected as helices by both methods. The similar β-sheet F1-scores of 0.73 (DeepSSETracer) and 0.69 (Emap2sec+) agree with visual inspection of similar detection overall with minor difference in the β-sheet area, in which false negative exist for Emap2sec+ (left red arrow of Figure 3D).

The weighted average of all 28 test cases shows that Emap2sec + detects helices slightly better than DeepSSETracer, with 0.77 and 0.72 residue-level F1-scores respectively (Table 2). Results also show that DeepSSETracer detects β-sheets much more accurately than Emap2sec+, with weighted average residue-level F1-scores of 0.65 and 0.42 respectively. The comparison suggests that each of the two methods has its own strength in detection of one of the two types of secondary structures for cryo-EM component maps. The 28 test cases were selected so that they do not share enough sequence identity or structural similarity with any chain of the training set used for DeepSSETracer. Since the 28 cases were not compared with the training data used in Emaps2sec+, they are not guaranteed to be significantly different from all the training data of Emap2sec+. Nevertheless, there has not been study about the effect of evaluation results when a test case shares sequence or structural similarity with a training data for the secondary structure detection problem.

The design of DeepSSETracer and Emap2sec+ are different, and this is reflected in different architecture of the network, training data, and training process. DeepSSETracer is designed for detection of secondary structures in component maps, such as those individual chains rather than the entire entry of a cryo-EM map, which often contains many chains. The training and testing for DeepSSETracer are based on component maps. Emaps2sec+ is trained and evaluated with entire entries of cryo-EM maps (Wang et al., 2021). The test here using the 28 component maps shows an aspect of performance when Emap2sec+ is applied to component maps, and it does not represent its performance on entire maps. The detection results were evaluated using residue-level F1-scores for individual classes, such as helix and β-sheets, because it is a stable performance measure at the residue-level. In DeepSSETracer, three class labels are defined, helix, β-sheet, and background. Note that the background class contains all other voxels that include those at the loop area and at the empty area without much molecular mass. The definition of class labels in Emap2sec+ is different in terms of how background is handled. It has a separate other class for molecular regions not in a helix, a β-sheet, and a nucleotide. The difference in class definition prevents from the comparison using Q4 score that was measured for Emap2sec+ (Wang et al., 2021). Even though the overall performance of all classes is not directly comparable, performance of individual classes can be compared using residue-level F1-scores for helix and β-sheet respectively.

## Materials and Methods

### Data Selection

All cryo-EM density maps were downloaded from EMDB (https://www.ebi.ac.uk/pdbe/emdb/) with a requirement of resolution between 5–10 Å and a corresponding atomic structure available in PDB. A density map is often associated with multiple chains of one or more proteins, but individual chains were used in training and testing. The atomic structure of individual chains of proteins were used as the envelopes to extract the density region of the chains in Chimera using a 5 Å radius parameter. Since it is common to see multiple copies of the same sequence in a cryo-EM density map, duplicated copies in each protein were removed from the pool to reduce training bias. Needleman-Wunsch algorithm was used to align a pair of sequences, and near identical chains (i.e., with more than 70% sequence identity) in the same protein were removed.

Since various levels of image quality exist among density maps and within the same map, a screening was performed to exclude chains with poor fit between the atomic structures and their corresponding component density maps. The cylindrical fit of helices was used as an estimate for the fit of the entire chain (Sazzed et al., 2020). More specifically, an F1 score was calculated from the fit for each helix in a chain, and the average F1 score over all helixes of a chain was subsequently computed and used to derive three bins of data representing top three levels of fit. A data set containing 1,382 chains from the top three bins was derived by the cylindrical fit. Each data point contains an atomic model of a chain and its corresponding cryo-EM component map. The data set was randomly partitioned into training (1,216 chains), validation (101 chains), and initial test set (65 chains). In order to ensure that test data are sufficiently different from the training data, additional screening was conducted. Firstly, those chains with unknown sequences were eliminated from the initial test set. Note that some chains have “UNK” marked as amino acid IDs in the PDB file, even though atomic coordinates were provided for the backbone of the protein chain. For the remaining chains in the test set, chains with a higher than 35% sequence identity with any of the chains in the training set were removed. To make sure that the test set is sufficiently different from those chains in the training set with unknown sequences, TM-align was used to compare structural similarity. A TM-align score of lower than 0.5 was required for at least one of the two TM-align scores that are normalized respectively for the lengths of the two sequences. The final test set contains 28 chains, each of which is sufficiently different from any other chain in the test set and from every chain in the training set through screening by either sequence identity or structural similarity.

After data screening, a total of 1,345 component density maps were partitioned into three disjoint subsets, used for training (N = 1,216), testing (N = 28) and validation (N = 101), respectively. Among the 1,216 chains in the training set, only 90 structurally unique chains exist, if a unique chain refers to one that has at least one of the 2 TM scores below 0.5. In an extreme case, a chain with a few helices may have a high TM score when comparing with a large chain (with many helices) if the score is normalized by the shorter length of the chains. However, the other TM score that is normalized by the longer chain is small in this case. For this reason, we required at least one of the 2 TM scores to be small for two structurally different chains. Our data selection process suggests that although many chains are available in the data, the number of unique chains is small in our top three bins. Note that structurally similar chains may have significant difference in two images, particularly in two maps with large quality difference. However, it is easier to measure uniqueness using sequences or structures than using images. Each density map is resampled to 1 Å per voxel. STRIDE was used to annotate secondary structures, for which H, G and I were considered as helix residues and B, b and E were considered as β-sheet residues. Voxels within 3 Å from Cα atom of the helix and β-sheet residues were labeled as helix voxel and β-sheet voxels, respectively. The rest of voxels were labeled as background.

### Neural Network Architecture

Since there is large variation in the size among protein chains, it is desired to have a neural network that accepts inputs in varying size. We employed a neural network architecture of end-to-end convolution operations, adapted from the 3D U-Net proposed in (Çiçek et al., 2016). This architecture naturally handles input density maps of different sizes by making a prediction, i.e., helix, β-sheet or other for each voxel in a map. More precisely, the input of the network is a 3D tensor (*x*-by-*y*-by-*z*) representing a density map and the output is a 4D tensor (*x*-by-*y*-by-*z*-by-3) providing the predicted probability associated with the three classes for each voxel in the input.

There are in total five composite layers in the network, each of which consists of two consecutive sets of operations. Both sets have in sequence the convolution, batch normalization and Relu nonlinearity operations. The third layer is the bottleneck layer, which divides the network into a down-sampling path (from the input to the bottleneck layer) and an up-sampling path (from the bottleneck layer to output). Consecutive layers are connected by a dropout operation followed by a max pooling in down-sampling or a transpose convolution in up-sampling. The network contains total 6,142,723 trainable parameters, with a receptive field, 35 × 35 × 35, for each voxel in the prediction. For detailed information and an illustration of the network, please refer to our previous work (Deng et al., 2020).

### Network Training

The aforementioned network was implemented and trained using the PyTorch framework with a loss function that combines cross-entropy loss and dice loss. To address the class imbalance (i.e., overwhelmingly more “other/background” voxels than either helix or sheet voxels), the cross-entropy was weighted inversely according to the class distribution in the training subset. Adam optimizer (Kingma and Ba, 2015) was used to optimize learnable weights in the network; while the hyper-parameters, e.g., dropout rate, step parameter in weight decay were optimized via grid search.

We trained the network with a curriculum, in which the difficulty of the learning task was gradually increased during the training process. Such a learning curriculum has been found effective in producing models with better performance than those obtained without using any curriculum by applications in other domains (Amodei et al., 2016; Jesson et al., 2017) and our previous work on protein secondary structure detection (Deng et al., 2020). More specifically, we divided the training process into three consecutive phases, starting from phase one with using only examples from Bin 1 followed by phase two using examples from Bin 1 and Bin 2 and then phase three using examples from all three bins. To further prevent forgetting what has been learned in an early phase during a later training phase, we implemented a variant of the technique referred as the gradient of episodic memory (GEM) proposed by Lopez-Paz and Ranzato (2017). In a nutshell, we randomly chose five examples used in previous training phase(s) to adjust the gradient in later phases to prevent forgetting. For detailed implementation of this method, please refer to our previous work (Deng et al., 2020), which also provides detailed comparison of three different learning curricula.

### DeepSSETracer Bundle Organization

**FIGURE 4:**
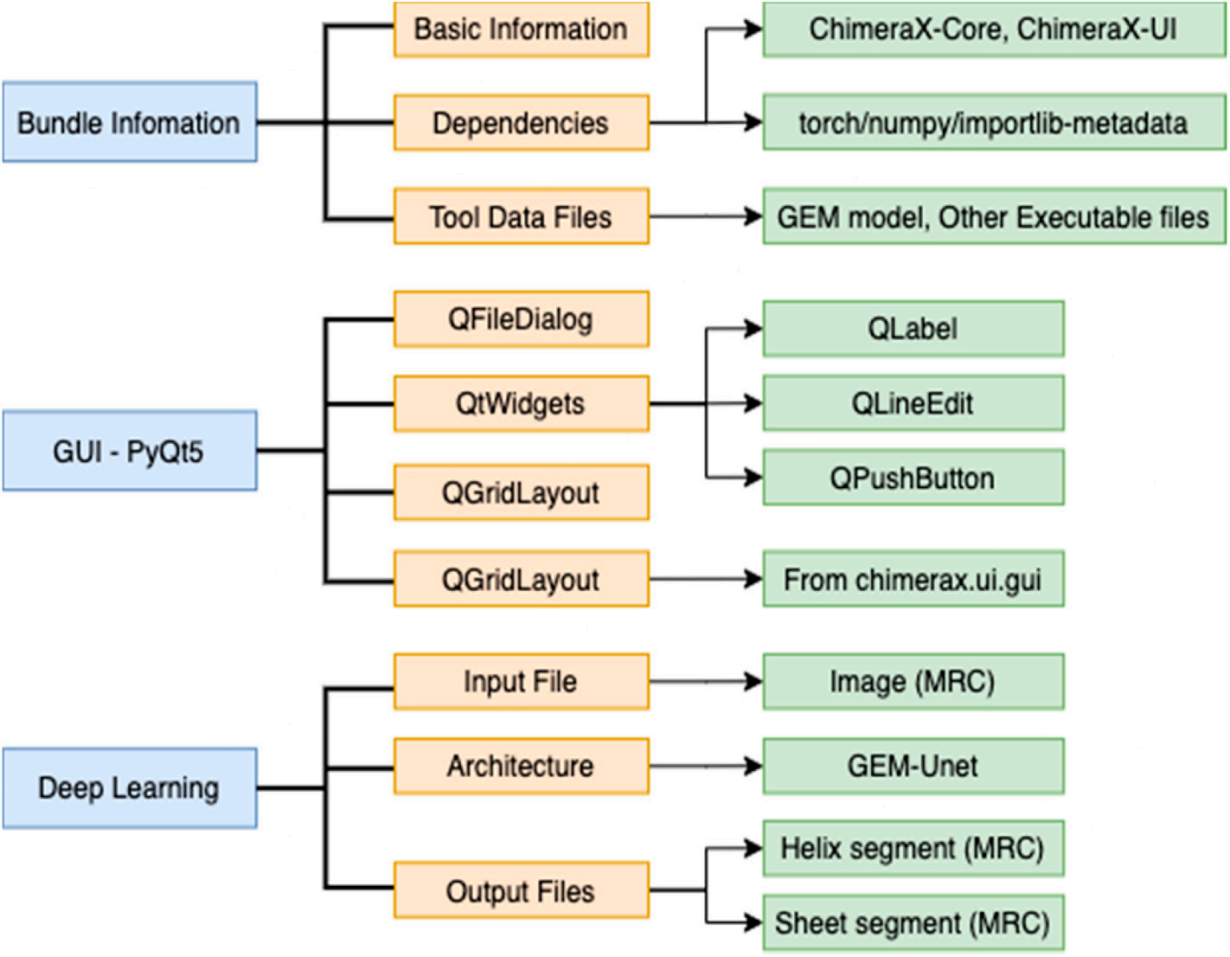
DeepSSETracer 0.1 bundle diagram.

The development of DeepSSETracer uses organization suggested in ChimeraX tutorial (https://www.cgl.ucsf.edu/chimerax/docs/devel/tutorials/introduction.html). Dependencies were defined in the bundle information file in xml format (Figure 4). This file defines the required software libraries, such as PyTorch, with specific versions. A main advantage is that a user does not need to install underlying platforms, since this process is automatically handled once required libraries are specified. The Graphic User Interface of DeepSSETracer was created using PyQt5 framework. A model previously trained using GEM-Unet architecture was provided in the bundle. A wheel file was generated to package the bundle information, the GUI-PyQt5, the Deep Learning architecture, and model (Figure 4).

## Conclusions

As large number of atomic structures and density maps are accumulated for molecular data, many computational methods are built on deep learning frameworks, such as Tensorflow and PyTorch. Although deep learning methodologies have been shown with improved performance in many problems, making the methodologies available to users without extensive computational knowledge is needed. We propose a software bundle, DeepSSETracer 0.1, for wrapping a pre-trained GEM-Unet model and the CNN architecture in a *Python* wheel for a user to install in ChimeraX. To our best knowledge, this is the first bundle reported for ChimeraX with a deep convolutional neural network. We showed the effectiveness of the integrated bundle in ChimeraX for secondary structure detection from cryo-EM component maps. It is fast, about 6 s, for a 5-layer U-Net deep learning architecture to predict secondary structures of proteins from a cryo-EM component map with 446 amino acids. However, the step to initialize the image data for the deep learning network takes about 27 s, and this step needs to be optimized. We evaluated the pre-trained GEM-Unet model and observed 0.72 and 0.65 averaged residue-level F1-scores for detection of helices and β-sheets, respectively, using a data set containing 28 test cases. Results from the comparison between DeepSSETracer and Emap2sec+ shows that DeepSSETracer detects β-sheets more accurately, and Emap2sec+ detects helices more accurately. We observed that using F1-scores that are measured at the residue level provides a sensitive and stable evaluation for the detection of individual classes, such as helix and β-sheet respectively.

## Data Availability

The original contributions presented in the study are included in the article/Supplementary Material, further inquiries can be directed to the corresponding authors.
